# The use of continuous surveys to generate and continuously report high quality timely maternal and newborn health data at the district level in Tanzania and Uganda

**DOI:** 10.1186/s13012-014-0112-1

**Published:** 2014-08-23

**Authors:** Tanya Marchant, Joanna Schellenberg, Stefan Peterson, Fatuma Manzi, Peter Waiswa, Claudia Hanson, Silas Temu, Kajjo Darious, Yovitha Sedekia, Joseph Akuze, Alexander K Rowe

**Affiliations:** Department of Disease Control, London School of Hygiene and Tropical Medicine, Keppel St, London, UK; Department of Public Health Sciences, Karolinska Institutet, Stockholm, Sweden; College of Health Sciences, Makerere University, Kampala, Uganda; International Maternal and Child Health Unit, Department of Women's and Children's Health, Uppsala University, Uppsala, Sweden; Ifakara Health Institute, Dar es Salaam, Tanzania; Malaria Branch, Division of Parasitic Diseases and Malaria, Center for Global Health, Centers for Disease Control and Prevention, Atlanta, USA

**Keywords:** Continuous survey, Quality improvement, Maternal and newborn health, Tanzania, Uganda

## Abstract

**Background:**

The lack of high quality timely data for evidence-informed decision making at the district level presents a challenge to improving maternal and newborn survival in low income settings. To address this problem, the EQUIP project (Expanded Quality Management using Information Power) implemented a continuous household and health facility survey for continuous feedback of data in two districts each in Tanzania and Uganda as part of a quality improvement innovation for mothers and newborns.

**Methods:**

Within EQUIP, continuous survey data were used for quality improvement (intervention districts) and for effect evaluation (intervention and comparison districts). Over 30 months of intervention (November 2011 to April 2014), EQUIP conducted continuous cross-sectional household and health facility surveys using 24 independent probability samples of household clusters to represent each district each month, and repeat censuses of all government health facilities. Using repeat samples in this way allowed data to be aggregated at six four-monthly intervals to track progress over time for evaluation, and for continuous feedback to quality improvement teams in intervention districts.

In both countries, one continuous survey team of eight people was employed to complete approximately 7,200 household and 200 facility interviews in year one. Data were collected using personal digital assistants. After every four months, routine tabulations of indicators were produced and synthesized to report cards for use by the quality improvement teams.

**Results:**

The first 12 months were implemented as planned. Completion of household interviews was 96% in Tanzania and 91% in Uganda. Indicators across the continuum of care were tabulated every four months, results discussed by quality improvement teams, and report cards generated to support their work.

**Conclusions:**

The EQUIP continuous surveys were feasible to implement as a method to continuously generate and report on demand and supply side indicators for maternal and newborn health; they have potential to be expanded to include other health topics. Documenting the design and implementation of a continuous data collection and feedback mechanism for prospective description, quality improvement, and evaluation in a low-income setting potentially represents a new paradigm that places equal weight on data systems for course correction, as well as evaluation.

**Electronic supplementary material:**

The online version of this article (doi:10.1186/s13012-014-0112-1) contains supplementary material, which is available to authorized users.

## Background

The last 10 years has seen maternal and newborn health gain momentum as a priority public health issue [1]. Although some important gains have been made, mortality rates remain high in many countries [2]. A review of evidence demonstrated that in high mortality, low income settings, interventions that save lives can be delivered at primary level health facilities, and centre around timely care seeking by women, the adoption of behaviours by families and health workers, and availability of low cost, low technology drugs and equipment [3]. In theory, it should be a simple process to realise the potential of these interventions, but in practice, the coverage of life saving interventions remains too low [4], and data for tracking progress remain scarce [5].

The lack of high quality timely data to support evidence-informed decision making presents an important challenge to improving survival for all in low income settings [6]-[8]. Existing large-scale data collection approaches such as the Demographic and Health Survey (DHS) [9], the Multiple Indicator Cluster Survey (MICS) [10], and the Service Provision Assessment (SPA) [9] provide high quality estimates for a broad set of indicators that are an invaluable resource for understanding health in a country at one point in time, and for tracking progress across time and place, but they have three important limitations. First, the periodicity of these surveys is usually once every two to five years, meaning that program managers have to wait a long time to assess progress of scale-up strategies, or to consider actions for course correction. The issue of periodicity is further exacerbated by temporality in that data collected is retrospective, sometimes reflecting events up to five years in the past. Second, estimates rarely go below the regional level and thus provide district health managers with little high quality, timely population data with which to set priorities or benchmark progress. Third, existing large-scale survey models cannot readily link indicators on population level health to indicators on healthcare availability and quality [11]. Health information system (HIS) data generated at individual health facilities and aggregated up to district, regional and national levels have potential for supporting decision making, but are limited to reporting on health service users, not population level statistics. But, currently, these data are of poor quality in the majority of high mortality settings and are not systematically used to manage, course-correct, and maximise the potential of health programmes [12].

In 2009, continuous surveys (as part of a larger, continuous quality improvement strategy) were proposed as a possible approach to apply high quality methods for data collection such as those applied by DHS, but adapted in ways that fundamentally change the survey outputs, particularly in terms of reporting intervals and unit of geographical disaggregation [13]. Continuous surveys are conceptualised as occurring at the national level, with national point estimates for a broad range of indicators several times per year (for input, output, and outcome indicators), and with sample sizes that have reasonable precision for national and regional level analysis annually. Indicators at the sub-national or district level could be calculated even more frequently using small-area analysis techniques [14]. These more frequent, lower-precision, sub-national estimates could be used for program management and support a continuous quality improvement strategy [13].

Variations of the continuous survey concept have already been applied in different settings. For example, in Peru a continuous survey was implemented nationally for five consecutive years, 2003 to 2008, in lieu of one national DHS survey, primarily because there was strong demand for department (district) level indicator estimates, as well as national level estimates [15]. It was concluded to be broadly successful in its design and implementation, recommended to continue in Peru, and recommended for other countries that currently rely on large scale surveys at intervals over three years. In Malawi, sub-district continuous surveys were implemented during a period of rapid scale-up of malaria control efforts in 2010 [16]. The implementers reported that these surveys were feasible and affordable, had provided valuable information about where to target malaria control efforts, and were recommended as a promising district level monitoring and evaluation tool for short-term processes. More recently, continuous household and health facility surveys have been implemented in Senegal [17].

Here we describe the experience of implementing continuous household and health facility surveys at the district level in Tanzania and Uganda that are part of a quality improvement project for maternal and newborn health, called EQUIP (Expanded Quality Management using Information Power) [18]. To our knowledge, this is the first example of a high quality data collection method that was designed to be used prospectively for description, quality improvement, and evaluation in a high mortality setting: this potentially represents a new paradigm that places equal weight on data systems for course correction as well as evaluation.

The protocol for the EQUIP intervention is provided elsewhere [18]. In brief, EQUIP was implemented in southern Tanzania and eastern Uganda. A quality management intervention was implemented that simultaneously engaged with district health management teams, primary and secondary level health facility workers, and community volunteers to collectively take small, incremental steps to improve demand and supply side factors that limit maternal and newborn healthcare. A central component of the quality improvement has been that all teams were provided with locally generated, high quality, population based data generated by the continuous survey described here. Every quarter, in line with the cycle of quality improvement team meetings, continuous survey data were analysed and summary report cards created around priority health topics: teams were then presented with these report cards and encouraged to reflect on how their own behaviours - and the behaviours of people operating in their community, facility, or management context - could improve demand or supply side factors associated with these health topics. In both countries, one district received both quality management and continuous surveys (intervention districts) and a neighbouring district had only continuous surveys (comparison districts). The success of district and health facility level quality improvement teams is reflected using supply side indicators (health facility survey), together with population level coverage of interventions that represent a combination of both supply and demand (household survey). The success of community level quality improvement teams is reflected using demand side indicators (household survey).

In this report, we present the continuous survey methods, their rationale, and results from the first 12 months of survey implementation. To display strengths, limitations, and generalizability of the methods, we endeavour to provide all relevant detail [19]. Evidence on the effect of the EQUIP intervention will be reported separately after completion of the intervention period.

## Methods

### Survey objectives

The EQUIP continuous survey had a dual objective. First, to generate high quality data that could be synthesized at frequent intervals, and with minimal delays, into report cards that were of interest to quality improvement teams working to improve demand or supply side factors affecting maternal and newborn healthcare locally. And second, to generate data that were of sufficiently high quality to carry out a robust evaluation of the EQIUP quality improvement intervention.

### Study setting

Maps of the study setting in both countries are available in the paper by Hanson, *et al.*[18]. Both countries established a decentralized district health system in the 1980s, and the populations are served by a network of public health facilities. Maternal and newborn healthcare is predominantly provided through these government facilities, although some women also seek maternity care from a small number of private or NGO-led (largely Christian mission) health facilities.

Administrative information for the four districts is provided in Table 1. In Tanzania, the intervention district (Tandahimba) and comparison district (Newala) are contiguous and have an unpaved road network, some roads being very difficult to pass during the rainy season. Each district has a single hospital. The maternal mortality ratio in the Southern Zone of Tanzania was estimated to be 712 per 100,000 live births [20], and the neonatal mortality rate 31 newborn deaths per 1,000 live births [21].

**Table 1:** **EQUIP continuous survey sample selection after first year of continuous survey implementation (November 2011 to December 2012)**

Country	District	N. clusters to be surveyed during year 1	N. administrative units for sub-district reports	N. villages	Estimated population size	N. facilities surveyed at each of 3 facility censuses	N. health worker interviews completed
Tanzania	Tandahimba	120^1^	3 divisions	157	227,514	32/32	96
Tanzania	Newala	120	5 divisions	155	205,492	30/30	84
Uganda	Mayuge	120	4 counties	512	412,500	38/38	97
Uganda	Namayingo	120	2 counties	250	233,100	22/22	58

^1^During implementation, one cluster was missed from month 7 in Tandahimba district due to civil unrest in the area and 119/120 clusters were surveyed there.

In Uganda, the intervention district (Mayuge) and comparison district (Namayingo) are also neighbouring and accessed by predominantly unpaved roads. Mayuge, but not Namayingo, has a district hospital. Both districts have small island populations that were included in early EQUIP planning, but which were subsequently dropped from the quality improvement and continuous survey protocols because of the high financial and time cost of reaching them. The maternal mortality ratio in Uganda was estimated to be 438 per 100,000 live births, and the neonatal mortality rate 23 newborn deaths per 1,000 live births [22]. In both settings, approximately two-thirds of women delivered their last born child in a health facility.

### Continuous survey overview

EQUIP aimed to conduct cross-sectional household and health facility surveys using 24 independent probability samples of household clusters to represent each district each month, and to conduct repeat censuses of all government health facilities in each district. Each month, in each district, 10 household clusters (including 300 households) and 5 to 10 health facilities were surveyed. At the end of each 4-month round, in each district, the survey included 40 household clusters (1,200 households) and a census of health facilities (22 to 38 facilities, depending on the district [Table 1]). To allow for holidays, retraining, and data processing intervals, these 24 monthly household and facility surveys were planned for implementation over the 30-month EQUIP intervention period (November 2011 to April 2014). Field work was organised into six rounds of data collection, each round including four-monthly household samples and one complete health facility census (Additional file 1).

Using repeat samples in this way allowed data to be aggregated at intervals to track progress over time. For example, the percent of newborns having clean cord care could be tabulated with reasonable precision at the district level after each round of survey (four months), and tabulated at the sub-district level (division in Tanzania and county in Uganda) after every three survey rounds (12 months).

### Sample size calculations and justification

In both of the Tanzanian districts, with an estimated total fertility rate of 5.4 in 2010 [9], it was expected that 10 clusters of 30 households each would result in 38 interviews per district each month with women who had a live birth in the 12 months prior to survey in each district, or 152 women per district in each four-monthly survey round. In Ugandan districts, with an estimated total fertility rate of 6.2 in 2011 [9], a higher number of women with a recent live birth would be interviewed. In Table 2, an illustrative set of sample size calculations are provided for a range of indicators across the continuum of care from pregnancy to newborn.

**Table 2:** **Illustrative sample size calculations** ^**1**^ **for outcomes along the continuum of care from pregnancy to post-natal in Tanzania and Uganda**

Stage in the continuum of care	Indicator		Expected level in comparison group	Sample size (live births) in each group needed to find percentage point increases of^2^:		
				10	15	20
Pre-pregnancy	1	Used family planning prior to new pregnancy	0.40	597	267	151
Antenatal	2	Attended ANC 4 or more times (women who gave birth in the last year)	0.43	603	268	150
	3	Use of IPTp in pregnancy reported by women who gave birth in the last year	0.63	525	223	119
Intra-partum	4	Births in a health facility	0.42	602	268	151
	5	Caesarean sections	0.04	208	113	75
Immediate newborn care	6	Nothing put on cord	0.72	428	175	89
	7	Immediate breastfeeding	0.20	452	214	127
Postnatal care	8	Mother reported post-natal check within 2 days and 7 days of child birth	0.30	549	251	145
	9	Infant received post-natal check within 2 days and 7 days of children birth	0.40	598	262	145

^1^Kirkwood B. and Sterne J, *Essentials of Medical Statistics*. Vol. 2 edition. 2001: Wiley-Blackwell.

^2^Indicators across the continuum of care from pre-pregnancy to post-partum, assuming 80% power to detect differences for given percentage point increases between intervention and comparison groups, or over time. Adjusted for design effect (1.4) and for refusals (10%).

For the purpose of evaluation, our assumption was that an increase of between 10 and 20 percentage points would occur in indicators across the continuum of care over the 30-month period of intervention, depending in part on the topics being addressed by quality improvement teams. The continuous survey sample was calculated to have at least 80% power to detect small increases (fewer than 10 percentage points) between the beginning and end of the intervention period across the whole spectrum of maternal and newborn care; 10 percentage point increases each year of the intervention for the majority of indicators (for example, institutional delivery), and larger increases more frequently (for example, 20 percentage point increases in percent of newborns having clean cord care every four months of the intervention period).

### Selection of clusters, households, and respondents

In both countries, probability methods were applied to select 24 independent probability samples of 10 clusters from each district prior to the start of survey.

In Tanzania, clusters were drawn using sub-village (vitongoji) household lists that had been compiled by a previous project in the study area in 2007 [23]. For each district, sub-villages were listed from north to south, the number of households in each sub-village cumulated, and clusters selected with probability proportional to the total number of households in the district. In the event that the selected cluster had more than 150 households, standard segmentation methods were applied [24] by a household survey mapper who visited each selected cluster prior to the survey interview team. The mapper then listed all households in the selected cluster (or segment) and systematically selected 30 households from that list using a fixed fraction (total number of households in the sub-village divided by 30).

Village level population data were not available for Uganda, and only one of the two Ugandan districts had enumeration area population data, as districts were reformed in 2011. Therefore, clusters were drawn using a government generated parish level list that stated total number of households per parish (a parish represents a group of 4 to 10 villages). For each district, parishes were listed from north to south, the number of households within parishes cumulated, and parishes selected with probability proportional to the total number of households in the district. Within each selected parish, villages were listed, allocated a random number, and the village with the lowest random number selected as the household cluster. Villages are relatively small in Uganda and all households were listed by the mapper with no segmentation, then 30 households systematically selected from the village list using a fixed fraction.

A list of the different data domains captured by the EQUIP continuous household and health facility survey is provided in Table 3 and includes input, process, output and outcome indicators, along the continuum of care from pre-pregnancy to the first 28 days of life of the newborn [25]. A total of 100 indicators were defined (Additional file 2). Questionnaires were developed based on well-established sequences of questions as used in DHS [9], MICS [10] and SPA [9], and also drew upon earlier work in the study areas [26]. The project held consultative meetings at national, regional and district levels prior to launching the continuous surveys in order to engage with stakeholders about the type of data they considered to be useful for decision making in maternal and newborn health.

**Table 3:** **EQUIP continuous household and health facility survey interviews and their data domains**

Survey type	Respondent	Procedures	Data domain
Household survey	Household head (or representative)	Written consent.	Household characteristics; household listing.
		Interview.	
	All resident women aged 15-49^1^	Written consent.	Use of ANC if current pregnancy;
			knowledge of HIV/AIDS;
		Interview.	knowledge and use of F/P; pregnancy history since Jan 2010.
	Women with a live birth since Jan 2010	Interview.	Knowledge of danger signs; use of antenatal, delivery, postpartum and postnatal healthcare; coverage of interventions for mothers and newborns.
	Women with a live birth in the last 60 days	Interview.	Care seeking for sick newborns.
	All women aged 15-49 years^1^	Interview.	Perceived quality of healthcare provided.
Facility census survey	Facility in-charge	Written consent.	Facility characteristics; routine facility services provided, employment of staff; supervision.
		Interview and record review.	
	Nurse in the maternity ward	Observation and record review.	Availability on day of survey of equipment, drugs, vaccines essential to deliver life-saving interventions; extraction of HMIS data about deliveries in the last 4 months.
	Health staff member who assisted the last birth recorded in the maternity register at the facility	Written consent.	Cadre, training and experience; preparation of essential equipment for last delivery attended; behaviours during the last delivery attended; knowledge of key processes during the continuum of care.
		Interview and record review.	

^1^Eligible women in Tanzania were those aged 13-49 years, in Uganda those aged 15-49 years.

In both countries, all consenting household heads were interviewed about usual residents and household characteristics. Further, all resident women (aged 13 to 49 years in Tanzania and 15 to 49 years in Uganda) who gave consent were interviewed.

### Repeat governmental health facility census and interviews with health staff

The EQUIP intervention targeted all government health facilities in intervention districts (representing almost exclusively the providers of routine maternal and newborn care in the study area); thus a repeat facility census was implemented rather than a sample survey of facilities. To maximise temporal alignment of data, each facility census was completed in each of the four districts once every four months, consistent with the reporting interval for household data. To try to minimise selection bias of health staff, the maternity register at each health facility was reviewed by the facility interviewer, and the member of facility staff who conducted the last delivery was invited to complete the health staff interview. If that member of staff was not at work on the day of survey, then the staff member assisting the second to last delivery was invited, and so on until an available staff respondent had been identified (Table 3).

All households and health facilities were mapped using a handheld global positioning system (GPS) with a 3-meter accuracy of `etrex Garmin’ type to capture their geographical locations (coordinates). Coordinates were downloaded and linked by the data manager to corresponding continuous survey data of each household and facility using unique identification numbers.

### Continuous survey teams

Each country budgeted for one continuous survey team to cover two districts throughout the 30 months of data collection. Previous in-country experience suggested that one team of eight people (one mapper, four household interviewers, one facility interviewer, one team supervisor, and one driver/vehicle) could complete 20 clusters per month when each cluster comprised 30 modular household interviews, a health facility assessment, and interviews with health facility staff [27]. The recruited team supervisor in each country was educated to degree level and the interviewers educated to secondary or higher level. Selection of the survey teams prioritised applications from people with previous experience of large-scale survey work, and with family members living in the same region as the study.

The continuous survey teams were supported by other EQUIP staff. One part-time senior scientist led the method development, sample selection, writing of quality control protocols, and wrote analytical plans. In each country, one part-time data manager programmed the survey instruments, managed the digital data downloads, and ran frequency and consistency checks on each month of data completed. And in each country, the overall project coordinator met the survey team each month to discuss experiences from the field. Further details about routine quality control and supervision are provided below.

### Quality control and supervision

The teams were trained in-house for five days prior to survey, followed by a two-cluster pilot and review of all procedures. Questionnaires were pre-tested prior to training and further refined following this pilot. These were implemented in Swahili in Tanzania and Lusoga in Uganda, using personal digital assistants (PDAs) [28]. The use of PDAs facilitated a number of quality control mechanisms. First, questionnaires were programmed to prevent illogical skip patterns or responses. Second, cluster and facility identifying numbers were pre-programmed on the basis of geographical information, and interviewers were prompted to confirm their entries. Third, daily download of data from the PDAs to the team supervisor's laptop enabled routine frequency checks to be run each day of survey, and queries addressed immediately. This back-up process also provided a high level of data security. Fourth, the team supervisor had a re-interview module on the PDA (implemented in 3 of every 30 households) that was designed as a checking mechanism for key variables. After daily download of all data, the supervisor ran a programme that was written to compare re-interview responses with original interview responses for the same household identification number and report on differences. This report was then used by the supervisor to discuss with interviewers why differences may have occurred. Site visits by the EQUIP coordinator and data manager were conducted once a month. Finally, the survey team in each country attended a refresher training after every four-monthly round (teams decided on a one-day duration in Uganda, three days in Tanzania). To assure teams that the continuous data were being used, after every four-monthly round, they were provided with tabulations of the indicators arising from the data they collected.

### Data management and analysis for report cards

The data management processes for the continuous surveys were similar to those for conventional periodic surveys, although they required routine operational support throughout the duration of the survey in contrast to the one-off peak activity. Data were downloaded from PDA to supervisor laptop, and from laptop to CD each day of survey: data remained on all three media until transferred to the secure hard drive in the project office and backed up. Once secured and backed-up, data tables for each cluster were checked for completeness against a paper summary report written by the field supervisor. When all expected interviews had been transferred and confirmed by the data manager, data were anonymised. After every four-monthly round of data collection, a pre-written computer program in STATA 12 (StataCorp LR, College Station, Texas, US) was run by the data manager to produce routine tabulations of all indicators, adjusting for clustering using the .svy commands. These tabulations were compiled in a district level report, and subsets of tabulations prepared as report cards. The concept behind the report cards was that individual teams would be motivated by seeing a health topic illustrated by data from their own locality. Importantly, both demand and supply side indicators would be displayed so that community volunteers, health facility staff, and district health management could consider which behaviours they wanted to change in their communities and could begin to think about ideas for achieving those changes. By having regular access to high quality data through repeat report cards, these teams could frequently update their knowledge and track any evidence of change. The process from finishing a round of data collection to producing tabulations that could be shared as report cards for quality improvement typically took four to six weeks. The process for developing report card content was initially led by EQUIP study staff who had pre-identified priority topics for quality improvement teams to address (for example, birth preparedness during antenatal care), but as the intervention proceeded, this increasingly involved consultation with the quality improvement mentors to tailor report cards to the health topics that teams themselves identified as being a priority. For evaluation of the EQUIP intervention effects, small-area analysis techniques [14] will be applied to further explore sub-district and temporal variations in demand and supply side improvements in maternal and newborn healthcare.

### Ethics

Ethical clearance was obtained from the local and institutional review boards from Ifakara Health Institute through the Commission for Science and Technology, (NIMR/HQ/r.8a/Vol.IX/1034), the Uganda National Council of Science and Technology and London School of Hygiene and Tropical Medicine (LSHTM) ethical clearance No 5888. Advocacy meetings were held at the start of the project with district and sub-district authorities, and repeated in Tanzania toward the end of the 12-month period. Communities and health facilities were informed about the survey one day prior to interview by the mapper who used information sheets in the local language. Written, informed consent to participate was obtained from each household head, woman, facility in-charge, and health staff interviewed.

## Results

### Sample selection and interviews completed

The first three rounds of data collection (120 clusters per district) took one month longer than the expected 12 months to complete in each country: November 2011 to December 2012 in Tanzania, and December 2011 to February 2013 in Uganda. In each district, 120 clusters of 30 households (10 clusters each month) were surveyed, with the exception of Tandahimba, where one cluster was missed due to civil unrest in the study area (Table 1). During the same period, all government health facilities were surveyed three times. The goal to interview one health facility staff member about a recent delivery at each facility was only realised in Tandahimba district; in Mayuge district, just 97 health staff interviews were conducted during 114 health facility interviews (Table 1). Missing interviews occurred most frequently in smaller facilities that were often staffed by a very small number of healthcare workers.

Completion of household interviews in all four districts was consistently high throughout the first year of survey when the goal was to survey a total of 3,600 households per district. The percent of sampled households with a completed household interview was 96% in Tanzanian districts, and 91% in Ugandan districts (Table 4). The percent of resident eligible women interviewed was 89% in Tanzania and 87% in Uganda (Table 4).

**Table 4:** **EQUIP continuous household survey interviews completed by district and by four-monthly round of implementation**

District	Round number	N. consenting households^1^	N. resident women aged 15-49 yrs^2^	N. (%) women interviewed^3^	N. women with a live birth in last 12 months
TANZANIA					
Tandahimba	1	1,135	1,195	1,027 (86%)	110
	2	1,144	1,228	1,108 (90%)	135
	3	1,157	1,175	1,061 (90%)	149
	Year total	3,436	3,598	3,196 (89%)	394
Newala	1	1,156	1,052	928 (88%)	91
	2	1,165	1,158	1,047 (90%)	128
	3	1,173	1,098	1,004 (91%)	155
	Year total	3,494	3,308	2,979 (90%)	374
UGANDA					
Mayuge	1	987	1,164	897 (77%)	225
	2	1,143	1,381	1,218 (88%)	272
	3	1,116	1,287	1,103 (86%)	206
	Year total	3,246	3,832	3,218 (84%)	703
Namayingo	1	1,047	1,250	1,035 (83%)	251
	2	1,130	1,336	1,207 (90%)	249
	3	1,116	1,261	1,125 (89%)	185
	Year total	3,293	3,847	3,367 (88%)	685

^1^Target number of households each four-monthly round was 1,200; target number of households per district in the first year was 3,600.

^2^Eligible women in Tanzania were those aged 13-49 years, in Uganda aged 15-49 years.

^3^N of eligible women interviewed, expressed as a percentage of resident eligible women.

### Generating report cards for quality improvement teams

Point estimates for indicators (Additional file 2) were tabulated after each round of data collection and entered into district-level reports together with their 95% confidence intervals. These reports were then discussed by quality improvement mentors who selected a smaller subset of indicators that related to the current topics of interest for the quality improvement teams working at different levels in the intervention districts. The development of report card format for each level of quality improvement team involved considerable piloting and consultation initially to determine preferred format and content (Additional file 3). However, once format had been established for each level, the interval between completing a four-monthly round of data collection and delivering a report card was typically four to six weeks. Contrary to the initial expectation of EQUIP staff, community level quality improvement teams showed considerable appetite for these report cards, as did facility and district level quality improvement teams, as evidenced by their repeat requests for new and updated report cards as the first year of implementation proceeded.

In addition, at the end of the first three rounds, the full indicator set was calculated using the aggregated 12 months of data, considerably reducing the width of confidence intervals around all point estimates. These estimates were recorded in a report and shared with the intervention district health management team during a meeting attended by the EQUIP data manager who could answer questions about data sources, as well as the EQUIP quality improvement lead. A similar report was generated for the comparison area district health management team but was not supported by a facilitative meeting that discussed the potential utility of the report for decision making.

### Continuous survey teams

In Tanzania, the survey team lived in the main town of Tandahimba district, using an EQUIP satellite office as a base. They were paid an annual salary according to institutional rates at Ifakara Health Institute. In Uganda, the survey team lived in Mayuge town, which is not located in either study district but is where the EQUIP office is based. They, too, were paid an annual salary according to Makerere University pay scales. In both countries, the team travelled to survey sites each day Monday to Friday in a project vehicle. Hours of work varied at their discretion according to distance to the cluster and season. For example, during cashew nut harvest in Tanzania, the team often chose to leave the project office very early so that they arrived at the selected cluster before women left for the fields at around 7.30 a.m.; in this way, they calculated that they spent less time toward the end of the day waiting to interview women who had not yet returned from farms. After the first year of implementation, in Tanzania, one household interviewer had left the eight-person team due to finding alternative employment. No one left the Ugandan team, but sadly a facility interviewer died from a cause unrelated to her work and needed to be replaced.

## Discussion

The objectives of the district level continuous household and health facility survey were met and surveys implemented as planned in Uganda and Tanzania, with few deviations. At the time of writing, 12 of 24 months of survey have been completed (in 13 calendar months), with completion rates comparable to those of other high-quality surveys. Every four months, the data were synthesized into report cards to support the decision making of quality improvement teams working at the levels of community, health facility, and district health management; the survey is also on track for evaluation of the EQUIP quality improvement innovation.

### Rationale for the continuous survey model in the context of quality improvement

Core to the design of the EQUIP continuous survey model was to place equal weight on a data system that could be used for prospective course correction, as well as for evaluation (results to be reported separately). Quality improvement teams in intervention districts were provided with report cards of synthesised survey data about topics that concerned them - whether the topic was new and teams wanted to understand the existing population or health facility level situation, or whether the topic had already been worked on and teams wanted to track changes in outcomes to assess the extent to which changes led to improvements. These report cards were produced once every four months showing evidence at the district level, and also once every year disaggregating evidence to the sub-district level.

The EQUIP continuous survey included both households and health facilities in each district. This was important for two reasons. First, increasing the coverage of life-saving interventions along the continuum of care, one of the core evaluation goals of EQUIP, required population level coverage (household survey) data for measurement. However, some of the life-saving interventions for mothers and newborns cannot be captured using household data alone [11]. For example, skilled attendance at birth is typically reported as a maternal health coverage indicator from household surveys but active management of the third stage of labour, a key life-saving behaviour performed by the skilled birth attendants, cannot be estimated from household interviews because women themselves may not remember - or may not understand - the life-saving behaviour. Instead, EQUIP asked women about skilled attendance at birth (household survey), and asked skilled attendants about the behaviours they carried out at the last birth they attended (health facility survey), and linked their responses at the level of the cluster. Second, improving health facility quality to provide quality care to mothers and newborns, from both perceived and readiness perspectives, was another core goal of EQUIP. Again, this required population level information from women about their perceptions of health facility quality, and facility level information about the availability of quality services.

The cost of implementing a continuous survey linked to quality improvement will be reported elsewhere (Manzi, forthcoming), but early analysis of costs incurred during data collection, data management, and supervision of the continuous survey in Tanzania indicated a per-household cost of US$15 in the first year of implementation. Meanwhile, a number of lessons have been learned about continuous data collection, and about continuous reporting of information, broadly summarised here under lessons about implementation and lessons about data use.

### Lessons about continuous survey implementation

1. Continuous surveys require continuous survey management. The one-off intense activity normally associated with cross sectional surveys was still present before the launch of the survey, but implementation of a survey over a long period of time needed sustained inputs and systems that could be completed by people working routine hours (for example, to check and maintain data quality, and to summarise data outcomes). One benefit of the requirement for such long-term sustained input has been to raise the profile and professional development of the data teams.
2. Applying repeat probability sampling methods to a district level sampling frame was essential in order to obtain representative samples each month, but resulted in some villages being selected more than once as the continuous survey proceeded: four different sub-villages were sampled two times in the first year of survey in both Tandahimba and Newala districts; 17 different villages were sampled twice, and 2 different villages were sampled three times in Namayingo district; 14 different villages were sampled twice in Mayuge district. Because data were anonymized, it was not possible to know whether or not individual households were sampled repeatedly. Repeat sampling of villages never occurred in contiguous months. While returning to the same villages more than once over a period of 12 months was not problematic in itself, the survey teams needed guidance in how to explain the sampling method to community members. It should be noted that a continuous survey implemented over a larger geographical area would be far less likely to experience this issue.
3. Although the questionnaire content was based on pre-existing multi-country survey tools, developments in definition of indicators (particularly for the relatively new field of newborn health) required that questionnaires be kept up to date while maintaining internal consistency so that different rounds of data collection would be comparable. While this was a design challenge, it was also an advantage to be able to add indicators to the survey as new priorities were identified, for example the life-saving newborn intervention `kangaroo mother care’ implemented at the community level.
4. The use of PDAs was important to facilitate the potential of continuous surveys to be timely in their output - but any changes in content of the questionnaire required reprogramming of tools and thorough retesting prior to implementation. Thus, having a data manager who could also programme the survey tools was a distinct advantage. Having protocols for storing data on multiple formats until the data manager had checked and secured data (for example, data kept on the individual PDA and team supervisor laptop, and burned to compact disk) was important, and no data loss was experienced in the first year.
5. Surprisingly, our concern about retention of survey team members was not justified; this may in part be due to the relatively small geographical area of implementation that allowed the teams to have a permanent home-base. The teams developed strong camaraderie and cooperation: indeed they had time to iron out any personality clashes that can arise during an intense data collection period. The inclusion of a three week break period once every four months - allowing time for data synthesis and preparations for the next round - appeared to have worked well in this context.

### Lessons about continuous data use

The synthesis and use of continuous survey data by quality improvement teams needed considerable facilitation. People with strong quality improvement skills do not necessarily have excellent skills in data interpretation. Understanding the limits of both survey and facility-based data, feeling confident in presenting the data sources, and overcoming resistance to the idea that communities and health facilities would have an appetite for such data were all obstacles that needed to be addressed to realise the potential of continuous surveys to continuously report. The data collected included a large set of demand and supply side indicators in maternal and newborn health, but the indicators best suited to quality improvement were those that might be expected to change in a short period of time, a relatively small subset of the entire data collected. Further, quality improvement teams repeatedly requested more and more granularity in data synthesis, which the continuous survey could not provide at frequent intervals, necessitating that each quality improvement team collected their own monitoring data to track progress in their locality. Finally, while continuous survey methods address problems around infrequent periodicity of reporting, the issue of temporality when interpreting evidence remained. Our sample size was determined by the expected number of women who had a birth 0 to 12 months preceding survey, which meant that there was a delay between the implementation time period and the possibility of observing effects in the household survey data that measured behaviours up to 12 months in the past. As such, the household data could not reflect changes arising from the quality improvement innovation during the first year of data collection, a point that frustrated quality improvement teams who preferred to see changes in real time. This frustration sometimes created tension between the learning from monitoring data and the learning from the survey data. To address this, EQUIP tried to maximise the use of facility data in report cards, reflecting service provision in the four-month interval between repeat censuses. It should be noted that this issue of temporality also presents a complex analytic issue in that EQUIP cannot easily correlate health facility readiness (as assessed from a survey census during a four-month period) with the quality of care received by women who had a recent birth (as assessed from a household survey with a 0 to 12 month recall period).

## Conclusions

The two district continuous surveys in Tanzania and Uganda proved to be feasible using a single survey team in each country during the first year of implementation. The dual objectives of the survey design were met. An important innovation from the EQUIP method was the continuous reporting of data for course-correction and evidence-informed decision making at multiple levels. Current research agendas to improve health in high mortality, data poor settings increasingly focus on mechanisms to enhance the use of data for decision making by in-country actors at the same time as using data for international comparisons and evaluation. Documenting the design, methods and implementation [19] of continuously reporting high quality data contributes to the body of knowledge about what can work to support countries achieve global health targets.

## Authors' contributions

TM, JS, FM, PM, CH, SP and AR conceived of the continuous survey in the context of the EQUIP project. TM, JS, AR, and CH adapted the methodology and data collection instruments. YS, ST, DK and JA tested and refined the methods, and oversaw implementation of the work. TM and AR drafted the manuscript. All authors read and approved the final manuscript.

## Additional files

## Electronic supplementary material

Additional file 1: Planned timetable for EQUIP continuous survey teams in Tanzania and Uganda*.(DOCX 11 KB)

Additional file 2: Comprehensive List Of EQUIP Continuous Survey Indicators.(DOCX 17 KB)

Additional file 3: Examples of report cards for three levels of EQUIP implementation in Tanzania and Uganda.(DOCX 295 KB)
