# Supplementary material for: The use of continuous surveys to generate and continuously report high quality timely maternal and newborn health data at the district level in Tanzania and Uganda
Source: Implement Sci. 2014 Aug 23;9:112. doi: 10.1186/s13012-014-0112-1 (PMC4160540; doi:10.1186/s13012-014-0112-1)
Supplement: Supplementary file 1 — Additional file 1: Planned timetable for EQUIP continuous survey teams in Tanzania and Uganda*.(DOCX 11 KB) [file 13012_2014_112_MOESM1_ESM.docx]

Additional file 1. Planned timetable for EQUIP continuous survey teams in Tanzania and Uganda*

| Round number | Survey period  (4 months of data collection) | Rest and dissemination | Preparation for next survey period |
| --- | --- | --- | --- |
| 1 | 1 Nov11 – 28 Feb12 | 1-22 Mar12 | 23-31 Mar12 |
| 2 | 1 Apr12 – 31^st^ July12 | 1-22 August12 | 23-31 August12 |
| 3 | 1 Sep12 – 31^st^ Dec12 | 1-22 Feb13 | 23-28 Feb13 |
| 4 | 1Mar-30^th^ Jun13 | 1-22 Jul13 | 23-31 Jul13 |
| 5 | 1Aug-30^th^ Nov13 | 1-27Dec13 | 28-31 Dec13 |
| 6 | 1Jan-31^st^ Apr 14 | END OF SURVEY |  |

*Real dates adapted to allow for country specific events, including public holidays
