# Supplementary material for: The use of continuous surveys to generate and continuously report high quality timely maternal and newborn health data at the district level in Tanzania and Uganda
Source: Implement Sci. 2014 Aug 23;9:112. doi: 10.1186/s13012-014-0112-1 (PMC4160540; doi:10.1186/s13012-014-0112-1)
Supplement: Supplementary file 2 — Additional file 2: Comprehensive List Of EQUIP Continuous Survey Indicators.(DOCX 17 KB) [file 13012_2014_112_MOESM2_ESM.docx]

Additional file 2. Comprehensive List Of EQUIP Continuous Survey Indicators

The list below shows indicators categorised by utilisation, service quality, knowledge, behaviours and intervention coverage. Those measured from the community perspective (through the household survey) are marked ”HH” and those measured in the facility survey are marked ”Fac”. Indicators in bold font are represent defined primary indicators for measurement of EQUIP impact.

##### Utilisation

- 1. ANC coverage: attendance at least once during pregnancy (HH)
  2. ANC coverage: attendance at least four times during pregnancy (HH)
  3. Mean number of ANC visits to a health facility during last pregnancy (HH)
  4. **Institutional delivery (HH)**
  5. Skilled attendant at delivery (HH)
  6. Birth by Caesarian section (%)(HH)
  7. Post-natal check (2 days) for babies born at home (HH)
  8. Post-natal check (7 days) for babies born at home (HH)
  9. Skilled provider of first post-natal check-up (HH)

1.10 Post-partum check: Mother at 2 days (HH)

1.11 Post-partum check: Mother at 7 days (HH)

##### Service quality (reported)

- 1. ANC timing: Median pregnancy weeks at first visit to ANC (HH)
  2. Content of antenatal care: blood pressure(HH)
  3. Content of antenatal care: urine tested (HH)
  4. Content of antenatal care: HIV counseling (HH)
  5. Content of antenatal care: blood tested (generic - HH)
  6. Content of antenatal care: HIV test result received (HH)
  7. Content of antenatal care: syphilis test result received (HH)
  8. Content of antenatal care: birth preparedness (HH)
  9. Successful referral to higher care (HH)
  10. Median length of facility stay after last delivery (HH)
  11. Antenatal card has complete recorded information (HH)

##### Service quality (perceived)

- 1. Problems accessing health care for self (HH)
  2. Confident a provider will be available (HH)
  3. The facility had all the medicines she needed (HH)
  4. Complete information given about any treatments (HH)
  5. Facility workers talked politely (HH)
  6. Facility workers helpful (HH)
  7. Given enough time to tell the medical staff everything she wanted to (HH)
  8. Medical staff listened carefully to what she had to say (HH)
  9. Facility was adequately clean (HH)
  10. Facility had a toilet (HH)
  11. Facility toilets were in good condition (HH)
  12. Drinking water was easily available in the facility (HH)

##### Service quality (facility readiness to provide care)

- 1. Physical infrastructure for maternity care (Fac)
  2. Availability of consumable supplies (all facilities) (Fac)
  3. Full vaccination services (Fac)
  4. STD service commodities (Fac)
  5. Anti-infective drugs for maternity care (Fac)
  6. Full antenatal care commodities (Fac)
  7. Full routine delivery care commodities (Fac)
  8. Minimum equipment for assisted delivery (all facilities) (Fac)
  9. Full emergency obstetric services - hospitals (Fac)
  10. Full newborn care commodities (Fac)
  11. Family planning commodities (Fac)
  12. Days services provided(Fac)
  13. Services outside clinic hours: maternity (Fac)
  14. Kangaroo mother care available at facility (Fac)
  15. Trained providers: Essential Newborn Care/Post Natal Care (Fac)
  16. Trained providers: Clean and safe delivery (Fac)
  17. Presence of at least one nurse/midwife (Fac)
  18. Presence of at least one medical trained personnel (Fac)
  19. Absenteeism (Fac)
  20. Transport for obstetric referral: motorised (Fac)
  21. Accompanied referral (Fac)
  22. Supervision (Fac)

##### Service quality (health worker reported activity at last birth)

- 1. Staff member was able to prepare all essential items for the last delivery event (Fac, last event)
  2. Mean number of essential items prepared (Fac, last event)
  3. Staff member monitored labour using a partograph(Fac, last event)
  4. **Staff member practices active management of the third stage of labour (Fac, last event)**
  5. Staff member has assistance during delivery(Fac, last event)

##### Knowledge amongst women

- 1. Knowledge of at least one contraceptive method (HH)
  2. **Knowledge of women aged 15-49 about at least one danger sign that may occur during pregnancy (HH)**
  3. **Mean number of pregnancy danger signs stated (HH)**
  4. Knowledge of women aged 15-49 about warning signs which would prompt referral during delivery (HH)
  5. Mean number of delivery danger signs stated (HH)
  6. **Knowledge of women aged 15-49 about at least one danger sign in the newborn (HH)**
  7. **Mean number of newborn danger signs stated (HH)**

##### Knowledge amongst health care providers

- 1. Staff member has knowledge of focused antenatal care(Fac, last event)
  2. Mean number of aspects cited (Fac, last event)
  3. Staff member has knowledge of which pregnant women required a special birth plan(Fac, last event)
  4. Mean number of aspects cited (Fac, last event)
  5. Staff member has knowledge of appropriate action to take in event of heavy bleeding after delivery (Fac, last event)
  6. Mean number of aspects cited (Fac, last event)
  7. Staff member has knowledge of appropriate care for the low birth weight baby(Fac, last event)
  8. Mean number of aspects cited (Fac, last event)

##### Behaviour and coverage of interventions

- 1. Family planning prevalence rate (HH)
  2. Unmet need for family planning (HH)
  3. IPTp: coverage of at least two doses of IPTp (HH)
  4. IPTp: mean number of doses of IPTp (HH)
  5. ITN coverage women 15-49 (HH)
  6. Tetanus toxoid vaccination (protection) in pregnancy (HH)
  7. Use of gloves by delivery attendant (home births) (HH)
  8. Preparation of clean delivery kit (home births) (HH)
  9. Infants weighed at birth (HH)
  10. Neonatal tetanus immunisation (HH)
  11. Care seeking for newborns (HH)
  12. Asphyxia management (HH)
  13. Cord cutting (HH)
  14. Clean cord tie (HH)
  15. Clean cord care (HH)
  16. **Immediate breastfeeding (HH)**
  17. Exclusive breastfeeding (3 days) (HH)
  18. Skin to skin contact (HH)
  19. Thermal care – immediate drying babies (HH)
  20. Thermal care – delayed bathing>6hrs (HH)
  21. Thermal care – immediate wrapping (HH)
  22. ITN use by newborns (HH)
  23. Proportion of all deliveries that were spontaneous during the last 4 months (Fac)
  24. Proportion of all deliveries that were caesarean sections during the last 4 months (Fac)
  25. Proportion of all deliveries that ended in a live birth during the last 4 months (Fac)
