# Supplementary material for: The use of continuous surveys to generate and continuously report high quality timely maternal and newborn health data at the district level in Tanzania and Uganda
Source: Implement Sci. 2014 Aug 23;9:112. doi: 10.1186/s13012-014-0112-1 (PMC4160540; doi:10.1186/s13012-014-0112-1)
Supplement: Supplementary file 3 — Additional file 3: Examples of report cards for three levels of EQUIP implementation in Tanzania and Uganda.(DOCX 295 KB) [file 13012_2014_112_MOESM3_ESM.docx]

**Additional file 3: Examples of report cards for three levels of EQUIP implementation in Tanzania and Uganda**

1. Annex 3a. Mayuge District: Example of reporting to district managers on maternal and newborn health indicators
2. Annex 3b. Tandahimba District: Example of community level report card on sepsis prevention for one round of continuous survey data
3. Annex 3c. Tandahimba District: Example of facility level report card synthesising evidence on sepsis management for one round of continuous survey data


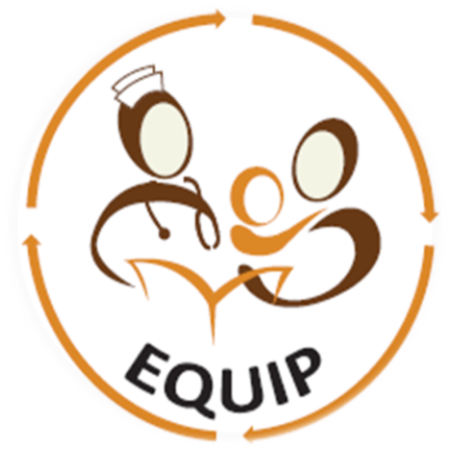


**EQUIP**

**Expanded Quality Management**

**Using Information Power to improve maternal and newborn health in Tanzania and Uganda**

As part of the quality improvement goals for maternal and newborn health services, the EQUIP project collects high quality data from households, health facilities and health workers between November 2011 and April 2014. Data are analysed once every four months in order to track progress towards improved quality of services for mothers and newborns. Results so far are presented here for **Mayuge**, and will be updated as new data is collected and analysed.

We can provide detailed breakdowns of each item on request.

The EQUIP project has completed the following data collection in the district:

**Table 1. Number of interviews completed as part of the EQUIP survey**

| Survey type | Nov 11 – Feb 12 | Apr 12 – July 12 | Oct 12 – Feb 13 | Mar 13 – Jun 13 | Aug 13 – Nov13 | Jan 14 – Apr 14 |
| --- | --- | --- | --- | --- | --- | --- |
| Households | 987 | 1143 | 1116 |  |  |  |
| Facilities | 38 | 38 | 38 |  |  |  |
| Health workers | 30 | 31 | 36 |  |  |  |

When we visited the **health facilities** in the district we made a record of the services available on the day that the survey took place. Vaccination services means that there was a working fridge or cold chain for vaccines, a sterilizer and needles or disposable needles and syringes. Anti-infective drugs for maternity care means that there was ampicillin, gentamicin, benzyl penicillin, and tetracycline in stock.

**Figure 1. Health facility readiness to provide care to mothers and newborns**

At the facilities **we asked the nurse or midwife** who had carried out the last delivery written in the record books about her experience. Preparation of all essential items means that the following had been prepared: gloves, disinfectant, gauze, cloth, scissors, ligature, oxytocin/ergometrine and eye ointment.

**Figure 2. Health worker experience at the last birth attended**

We also **asked the nurse or midwife** about knowledge of providing focussed antenatal care, what actions should be taken for a mother who starts to bleed heavily during or after childbirth, and what actions should be taken for a low birthweight baby. When asking these questions we waited for the staff member to tell us the answers she thought were correct, then calculated the average number of correct answers provided.

**Table 2. Mean scores for knowledge about (1)FANC, (2) about actions to be taken for women with heavy bleeding during or immediately after delivery, and (3)for low birth weight babies**

| Knowledge of: | Total possible: | Nov 11 – Feb 12 | Apr 12 – July 12 | Oct 12 – Feb 13 | Mar 13 – Jun 13 | Aug 13 – Nov13 | Jan 14 – Apr 14 |
| --- | --- | --- | --- | --- | --- | --- | --- |
| Components of focussed antenatal care (FANC)^1^ | 6 | 4.6 | 4.1 | 3.6 |  |  |  |
| Actions for women with heavy bleeding^2^ | 8 | 6.2 | 5.3 | 4.1 |  |  |  |
| Actions for low birthweight babies^3^ | 5 | 3.5 | 2.9 | 1.6 |  |  |  |

^1^FANC possible score was 6: minimum of 4 visits to ANC; birth planning; prevention of illness and promoting health; detection of existing illnesses and infections; teaching danger signs; promoting breastfeeding.

^2^Heavy bleeding possible score was 8: Massage the fundus; give ergometrine or oxytocin; begin IV fluids; empty a full bladder; take blood for haemoglobin testing or cross matching; examine the woman for lacerations; manually remove retained products; refer if necessary.

^3^LBW babies possible score was 5: keep baby warm; provide support to establish breastfeeding; monitor ability to breastfeed; monitor baby for at least 24 hours; ensure infection prevention.

**At households** we asked women who had a live birth in the 12 months before the surveys about their use of health services during pregnancy (ANC), birth (facility delivery) and checks on herself (PPC) and her newborn (PPC) within 7 days of the birth.

**Figure 3. Use of health care services for mothers and newborns**

**Table 3. Mean number of ANC visits made by women**

|  | Nov 11 – Feb 12 | Apr 12 – July 12 | Oct 12 – Feb 13 | Mar 13 – Jun 13 | Aug 13 – Nov13 | Jan 14 – Apr 14 |
| --- | --- | --- | --- | --- | --- | --- |
| Mean number of visits to ANC | 3.6 | 3.3 | 3.4 |  |  |  |

We also **asked each woman** about what happened at ANC, including whether her blood pressure was measured at least once, whether her urine was tested, whether she received a syphilis test result, and whether she was counselled about birth preparedness.

**Figure 4. Content of antenatal care checks**

**Each woman** was asked about her knowledge of danger signs in pregnancy, during delivery, or for the newborn. When asking these questions we waited for the woman to tell us the answers she thought were correct, then calculated the percentage of women who knew at least one danger sign at each stage.

**Figure 5. Knowledge amongst women of at least one danger sign during pregnancy, child birth, and for the newborn**

Finally, women were asked about coverage of interventions and behaviours that save newborn lives.

**Figure 6. Coverage of behaviours that save newborn lives**

| **Clean cord cutting, tying and cord care** | **Immediate breastfeeding and exclusive breastfeeding for first three days of life** |
| --- | --- |
|  |  |
| **Thermal care: immediate drying and wrapping of the newborn, delayed bathing by > 6 hours** | **Asphyxia management for newborns who experience difficulty breathing at birth** |
|  |  |

**Women in Tandahimba who delivered at home and prepared clean clothes for their delivery**

**24% of women living in Tandahimba who had a recent birth at home prepared clean clothes for their delivery**

**Women in Tandahimba who delivered at home and prepared gloves for her birth attendant to use during delivery**

**21% of women living in Tandahimba who had a recent birth at home prepared gloves for her birth attendant**

**Women in Tandahimba who delivered at home and prepared a clean cover to deliver on**

**31% of women living in Tandahimba who had a recent birth at home prepared a clean cover to deliver on**

**Women in Tandahimba who delivered at home and prepared cotton gauze for use during delivery**

**33% of women living in Tandahimba who had a recent birth at home prepared gauze for use during delivery**

**Women in Tandahimba who delivered at home reported that the birth attendant used gloves during the birth**

**92% of women living in Tandahimba who had a recent birth at home said the birth attendant used gloves**

**Women in Tandahimba who delivered at home reported that their baby’s cord was tied using a clean cord tie**

**70% of women living in Tandahimba who had a recent birth at home said their baby’s cord was tied using a clean cord tie**

**Women in Tandahimba who delivered at home reported that their baby’s cord was cut using a clean blade**

**94% of women living in Tandahimba who had a recent birth at home said their baby’s cord was cut using a clean blade**

**Women in Tandahimba who delivered at home reported that they put nothing on the baby’s cord after birth**

**98% of women living in Tandahimba who had a recent birth at home said they put nothing on their baby’s cord**

**Health facilities in Tandahimba had soap, disinfectant, and disposable gloves in stock on the day of survey**

**Health facilities in Tandahimba had a source of clean running water and an accessible toilet on the day of survey**

Dispensary Health Centre Hospital

**Health facilities in Tandahimba had sterile blades and clean cord ties for the newborn on the day of survey**

**Health facilities in Tandahimba had a functional sterilizer and a sharps box in place on the day of survey**
